# The Otoacoustic Emissions in the Universal Neonatal Hearing Screening: A Scoping Review Update on the African Data (2004 to 2024)

**DOI:** 10.3390/children12020141

**Published:** 2025-01-27

**Authors:** Stavros Hatzopoulos, Ludovica Cardinali, Piotr Henryk Skarzynski, Giovanna Zimatore

**Affiliations:** 1 Clinic of Audiology & ENT, University of Ferrara, 44121 Ferrara, Italy; sdh1@unife.it; 2 Department of Life Science, Health, and Health Professions, Link Campus University, 00165 Rome, Italy; l.cardinali@unilink.it; 3 Heart Failure and Cardiac Rehabilitation Department, Faculty of Medicine and Dentistry, Medical University of Warsaw, 02-005 Warsaw, Poland; piotrhskarzynski@gmail.com; 4 Institute of Sensory Organs, 05-830 Kajetany, Poland; 5 World Hearing Center, Department of Teleaudiology and Screening, Institute of Physiology and Pathology of Hearing, 02-042 Warsaw, Poland; 6 Department of Theoretical and Applied Sciences Applied Physics, eCampus University, 22060 Novedrate, Italy

**Keywords:** congenital hearing loss, newborn hearing screening, otoacoustic emissions, well babies, NICU, bilateral hearing loss

## Abstract

**Background:** The reported data on African universal neonatal hearing screening (UNHS) practices tend to be quite scarce, despite the developments in hearing screening the last two decades. The objective of this systematic review was (a) to identify the most recent (in a 20-year span) literature information about NHS/UNHS programs in Africa and (b) to provide data on the procedures used to assess the population, the intervention policies, and on the estimated prevalence of congenital hearing loss with an emphasis on bilateral hearing loss cases. **Methods**: Queries were conducted via the PubMed, Scopus, and Google Scholar databases for the time window of 2004–2024. The mesh terms used were “OAE”, “universal neonatal hearing screening”, “congenital hearing loss”, “well babies”, and “Africa”. Only research articles and review papers were considered as good candidates. The standard English language filter was not used, to identify information from non-English-speaking scientific communities and groups. **Results**: Data from 15 papers were considered, reflecting the neonatal hearing practices of nine African states. No country-wide NHS programs were reported. The various screening realities are implemented within big urban centers, leaving the residents of rural areas unassisted. For the latter, proposals based on tele-medicine protocols have been suggested. The data on HL prevalence are also incomplete, but the available data refer to rates from 3 to 360 subjects per 1000. These data cannot be taken at face value but within the small sample size context in which they were acquired. Regarding the causes of HL, very few data have been reported; consanguinity is the most attributed factor, at least in the Sub-Saharan African states. For the majority of the programs, no data were reported on hearing loss prevalence/incidence or on any strategies to restore hearing. **Conclusions**: The information on the African neonatal hearing screening are quite scarce, and it is an urgent need to convince audiologists from the African localized programs to publish their hearing screening data.

## 1. Introduction

Neonatal hearing screening (NHS) is an early hearing detection and intervention strategy, which aims to identify infants with potential conductive and sensorineural hearing deficits. Data in the literature strongly suggest that the early detection and rehabilitation of a hearing impairment are essential factors for the development of language and the relative social and cognitive skills [1,2]. At present, two clinical methodologies are available to conduct universal neonatal hearing screening (UNHS); these protocols are based on otoacoustic emissions (OAEs) or on automated auditory brainstem responses (AABR) [3,4]. The basic details on the impact of hearing screening to language development and the inner workings of the clinical screening protocols have been presented in a previous paper [5].

OAEs are a quick, non-invasive, and cost-effective technique. A tiny probe is inserted into the newborn’s ear canal, and its microphones produce certain transient stimuli (such as clicks, chirps, and tone busts). A short period of time, measured in milliseconds, is then recorded by the same microphones, which capture the acoustical echoes produced by the inner ear reflecting the stimulus energy [5]. This sort of technology has wide-ranging applications, extending to the detection of age-related hearing loss in adults [6] and in noise-induced hearing loss [7], where a new proposed protocol can identify hearing impairment much earlier than the conventional pure-tone audiometry and the classical TEOAE pass/fail screening criteria.

Even though hearing screening is considered an established clinical practice, NHS programs seem to face many implementation difficulties, as we have previously shown in a review of the European NHS data [5]. Extrapolating this information to states where economic and cultural difficulties are very present (i.e., the stigma related to a hearing loss) [8] is a challenge. Africa is a continent with numerous developing economies, and NHS information is seriously lacking in the literature. As early as 2008, Swanepoel and Storbeck [9] provided initial information on the UNHS-EDHI (early detection and hearing intervention) practices in Africa, with more emphasis in the activities of South Africa. Nevertheless, sixteen years later, Yoshinaga-Itano [10] reported that we still face a lack of screening outcome data (i.e., intervention policies) from African UNHS programs. That statement drove the impetus to discover all possible information about the hearing screening practices in African countries and summarize them within a scoping review.

In addition, the paper has sought suitable responses (when information was attainable from the literature) to the following five issues which are very fundamental for the EDHI strategies: (i) Which countries perform NHS/UNHS? (ii) What percentage of newborns are involved? (iii) How many present congenital deafness and more precisely bilateral deafness? (iv) What is the follow-up rate? (v) Which are the most common protocols and OAE technologies used to assess hearing?

## 2. Materials and Methods

We sourced scientific articles and reviews using the PubMed, Scopus, and Google Scholar search engines. As in the previous review on the NHS practices in Europe, we have selected a search time-span window of the last 20 years. The main reason for selecting this time window is that the majority of the centralized NHS screening programs [1,10] appear after the year 2000, aided by the fact that the screening technology became quite affordable, with the appearance in the market of portable automated OAE (AOAE) and AABR screeners.

The African continent is composed by 54 countries. In various contexts, Africa is being divided into the northern part and the Sub-Saharan part, which includes 50 states. In this review, we have considered the whole continent without any specific geographical divisions. We focused our investigation on the 84.45% of the total African population (1,532,073,577 as measured in 2024 and reported in [11]), in terms of the first 25 population-dense African states, which are shown in Table 1.

The literature search, conducted on November 2024, followed the PRISMA 2020 guidelines (the PRISMA website was visited in July 2024, see Table S1) and utilized the following 5 keywords and phrases (mesh terms): “OAE”, “neonatal hearing screening”, “congenital hearing loss”, “well babies” and “Africa”. Only research articles and review papers were considered as good candidates. The standard English language filter was not used, to identify possible information from non-English-speaking (i.e., French) scientific communities and groups. Papers related to NHS practices outside the African continent were not considered.

The quality of the material was dictated by several factors, with the publication being in a peer review journal being the cardinal one. Additionally, clearly stated methodologies and a rigorous application of the established screening protocols were also taken into consideration.

The selection criteria for the candidates of the search were based on (i) the origin of the paper (African group or not); (ii) the number of infants screened (large sample studies were preferred), and (iii) the most recent data per country (the most recent study/studies per country were selected).

The surprising fact about the lack of European screening data, we have previously reported in [5], was also repeated for the African countries. Therefore, the stringent criteria we had apply for the European states were lessened. Due to lack of information, we decided to include local studies even referring to small samples, to obtain a glimpse of the screening characteristics of the assessed population.

The PubMed, Scopus, and Google Scholar bibliographic data were considered. Two independent reviewers went over the available material (74 manuscripts), and the final number of eligible papers were distilled to 15 (related to 9 NHS realities, not UNHS, programs). The papers included a thesis dedicated to the status of NHS in the Sub-Saharan area.

**Figure 1:**
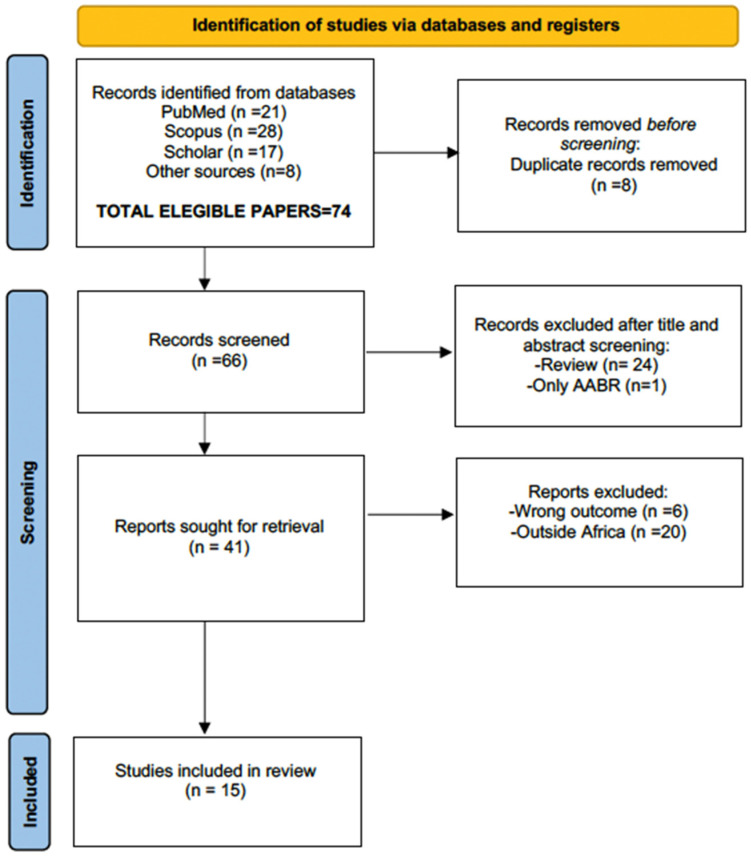
Flow diagram of literature search, according to PRISMA criteria (http://www.prisma-statement.org/, accessed 30 July 2024), with the steps followed in the manuscript selection procedure. After the application of the selection criteria, the initial 74 manuscripts were reduced to 15. Note: the “wrong outcome” voice implies that certain manuscripts were erroneously selected, for example providing data on onchocerciasis-associated epilepsy (OAE) and not on otoacoustic emissions.

The PRISMA flowchart process is reported in Figure 1, and the suitable papers for review are reported in Table 2.

## 3. Results

**Figure 2:**
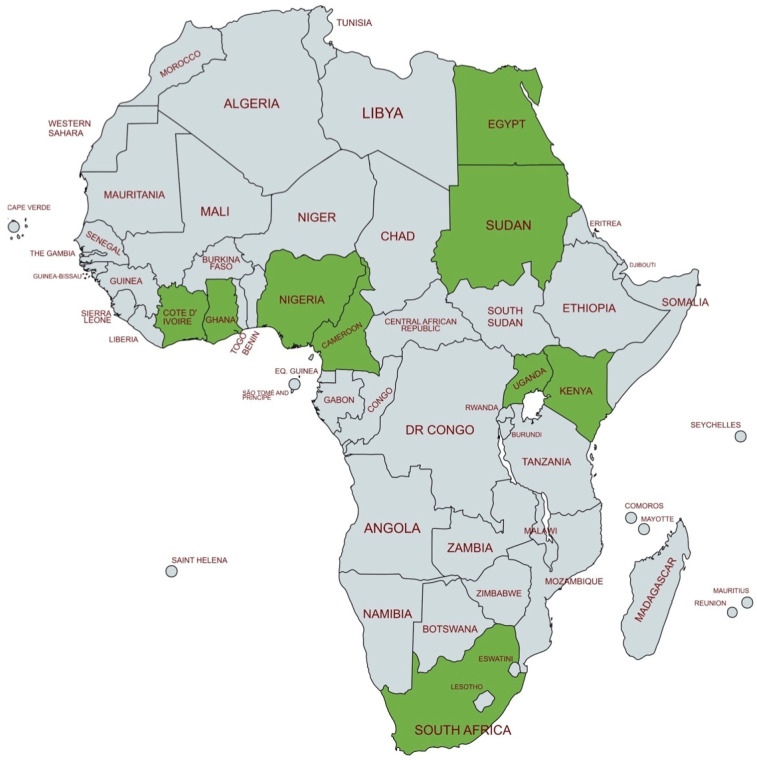
Political map of Africa showing the countries, in green, where NHS activities have been reported in the literature. These African states are reported alphabetically in Table 2.

The data were classified alphabetically, according to the country of origin, and the obtained information is summarized in Table 3, at the end of this section. Figure 2 depicts the African countries where NHS practices are reported.

### 3.1. Cameroon

There are no NHS data from Cameroon, but there is information on the hearing impairment status across various strata of the Cameroon society. Tingang et al. [12] report that the prevalence of hearing impairment (HI) in Cameroon ranges from 0.9% to 3.6% in population-based studies and increases with age. Environmental factors contribute to 52.6% to 62.2% of HI cases, with meningitis, impacted wax, and age-related disorders being the most common ones. Hereditary HI comprises 0.8% to 14.8% of all cases. In 32.6% to 37% of HI cases, the origin remains unknown. Non-syndromic hearing impairment (NSHI) is the most frequent clinical entity and accounts for 86.1% to 92.5% of cases of HI of genetic origin. Waardenburg and Usher syndromes account for 50% to 57.14% and 8.9% to 42.9% of genetic syndromic cases, respectively. No pathogenic mutation was described in the GJB6 gene, and the prevalence of pathogenic mutations in GJB2 gene ranged from 0% to 0.5%.

The prevalence of permanent hearing loss in Cameroon was found to be from 0.9% to 3.6% (9–36 per 1000), increasing with age.

### 3.2. Cote d’Ivoire

There is only one pilot study on this country dating back to 2009, which was conducted by Tanon-Anoh et al. [13] at the Abidjan, which is the economic capital of the country. The authors tested well babies (WB) and neonatal intensive care unit (NICU) residents (from 3 to 28 days), simulating a UNHS program. The testing protocol consisted by of a double TEOAE test in the first phase and clinical ABR, tympanometry, and OAEs in the second phase.

They reported the following: “1306 newborns, of a total of 1495, were successfully screened, giving a screening coverage of 87.4%. The average age was 4.5 days, with 5.85 days for the immunization group and 3.20 days for the neonatal unit group. After the second-stage screening, 48 (16.8%) were scheduled for diagnostic evaluation (45 from NICU and 3 from primary care centers). The overall referral rate for diagnostic evaluation was 3.7% (48/1306). Only 18.75% of those referred (9/48) returned for evaluation, and seven of them (77.8%) were confirmed with hearing loss”.

The prevalence of permanent hearing loss in this screened population was 5.96 per 1000 (7/1174 babies who completed the screening).

### 3.3. Egypt

There is no UNHS or NHS program in Egypt. The only information from the Egyptian hearing screening comes from the publication by Imam et al. [14] on the evaluation of targeted versus universal screening practices. The authors employed a rather small sample of 150 infants, including 50 well babies and 100 NICU residents. The NICU residents were divided into two groups: one with only one risk factor (50 infants) termed low risk and one presenting 3–4 risk factors (50 infants) termed high-risk. The testing protocol consisted by of a double TEOAE test in the first phase and clinical ABR, tympanometry, and OAEs in the second phase. Their findings support that a UNHS approach to screening is preferable to a targeted one.

Analytically, the authors report the following: “The most frequent risk factor was consanguinity (46%). In the well-baby population, 16% failed TEOAEs. In the NICU, 30% of the low risk and 38% of the high-risk groups failed TEOAEs. Regarding ABR, failed results were 12%, 10%, and 8% in the high-risk, low-risk, and healthy groups, respectively. Conclusion. The use of targeted screening would have missed 8% of neonates from the well-baby group who present permanent congenital hearing loss”.

The authors also speculate on the high estimates of the hearing impairment encountered in their sample, referring to other analyses, which have supported the presence of connexin 26 (Cx26) mutations and alterations in the southern Egyptian population [15]. Due to the small sample used in the study, the estimated incidence of hearing loss of 360 per 1000 is obviously erroneous, as it was based on selected cases and not on a rather large and randomized sample.

### 3.4. Ghana

According Ameyaw et al. [16], in Ghana, there is an established infant hearing screening, as part of its Early Hearing Detection and Intervention program. However, because of the lack of hearing health care specialists, especially pediatric audiologists, newborn hearing screening and other infant hearing services are accessible only at the Korle-Bu Teaching Hospital (KBTH) located in the Greater Accra Region of Ghana. Data in the literature regarding the EDHI program at KBTH are not available. The paper by Ameyaw et al. describes the results of applying tele-health technologies in neonatal hearing screening. The authors used a sample of 50 infants (aged 2–90 days) in the Brong-Ahafo Regional Hospital. The infants were assessed with a DPOAE protocol from a Bio-Logic AuDX device. The paper is focused on the technical aspects of the tele-health protocol.

Adadey et al. [17], in a paper related to the hearing impairment implications of GJB2 genetic screening in 2020, also report that there is a UNHS program in Ghana that is unavailable in most health centers across the country. For the authors, the failure to effectively establish the UNHS across the country could be due to a variety of reasons, including the high cost of testing, limited infrastructural capacity, and the lack of human resources to man the service.

### 3.5. Kenya

There is no NHS or UNHS running program in Kenya. The most recent data come from the paper of Ndegwa et al. in 2024 [18], describing a pilot UNHS program in the capital city of Nairobi. The authors report the following: “The screening coverage rate was 98.6% (9963/10,104). The referral rate for the initial screen was 3.6% (356/9963), the return rate for follow-up rescreening was 72% (258 babies out of 356) with a Lost To Follow-up rate of 28% (98/356). The referral rate of the second screen was 10% (26/258). All the 26 babies referred from the second screen returned for diagnostic hearing evaluation and were confirmed with hearing loss, yielding a prevalence of 26/9963 = 3 per 1000”.

The 26 infants presenting hearing loss were 21 bilateral and 5 unilateral cases. The bilateral cases were composed by four mild, nine moderate, five severe, and three profound hearing loss cases. The paper does not offer any explanation on the causality of the hearing loss, and the authors do not report any intervention policies for the infants presenting hearing loss; although, in their paper, Figure 1 refers to hearing aid fitting after an ENT consultation. Despite the fact the project was funded by MEDEL, no data on any cochlear implant recipients are presented.

### 3.6. Nigeria

There is no active NHS or UNHS program in Nigeria; although, a number of pilot studies by Olusanya et al. [19,20] have been reported in the literature.

In the first study [19], the authors reported that, of the 3676 infants enrolled, 52.4% were not born in hospital, and 71 (2.1%) were confirmed to have SNHL. For the 13 infants presenting bilateral hearing loss, 3 presented a moderate SNHL, 8 presented a severe SNHL and 3 presented a profound SNHL. One infant presented unilateral loss. Six of the infants with SNHL had a profile suggestive of auditory neuropathy/dys-synchrony. Data on any intervention policies (hearing aids, cochlear implants) are not reported.

The second study [20] was an attempt to evaluate the performance of two major tools in NHS, namely OAEs and AABR. The authors reported that 1745 (36.9%) infants completed both TEOAE and AABR. Of this group, 1060 (60.7%) passed both TEOAE and AABR (“true-negatives”); 92 (5.3%) failed both TEOAE and AABR (“true-positive”); 571 (32.7%) failed TEOAE but passed AABR (“false-positives”), while 22 (1.3%) passed TEOAE but failed AABR (“false-negatives”). No HL incidence is reported.

### 3.7. Sudan

There is no NHS or UNHS program in Sudan, and the only hearing screening report available is a paper from Kardman et al. [21], in 2023, on a project conducted in the capital of the country Khartoum. The authors assessed 736 NICU residents and 384 well babies over a period of 5 years, using TEOAEs and a clinical ABR. From the risk factors reported, consanguinity, ototoxic drugs, gestational age, and neonatal jaundice were the most reported. From the NICU infants, 16 did not pass the TEOAE screening test, and the authors reported some problems with 8 NICU infants who were scheduled to be retested. Only 8 infants were evaluated with an ABR and all presented bilateral hearing loss. The estimated prevalence in this group was reported as 10.8 per 1000, which is underestimated, since 50% of the referred subjects were not assessed by ABR. From the 384 well babies, 6 failed the TEOAE test. From those, four did not return for the clinical ABR, and the other two were verified with SNHL. The HL incidence of the well-baby group was reported as 5.2 per 1000, which is also an underestimate of the true incidence, since 66.6% of the referred infants were not assessed with ABR.

### 3.8. South Africa

According to Bezuidenhout et al. [22], the state of South Africa is the most developed in terms of hearing screening practices, but still, no formalized and standardized system of newborn hearing screening (NHS) exists at state-run hospitals in Johannesburg, South Africa (SA). Only risk-based hearing screening occurs, and this is also not systematic. We have identified four manuscripts referring to four different glimpses of NHS development in that country. From the oldest to the newest, these reports are as follows.

The first paper by Swanepoel et al. [23] explored the possibilities to conduct an OAE plus high-frequency tympanometry (1000 Hz) assessment on selected infants, in two private centers for a period of 5 months. The study did not report any HL incidence or any EDHI policies, but the authors reported that “Follow-up screening appointments were scheduled for 68 subjects (14% of screened sample). Only 40% returned for the second follow-up and 44% for the third follow-up”.

The second paper by Swanepoel et al. [24] refers to data from a retrospective study on 6241 infants, in a private hospital in urban Gauteng, South Africa. The authors reported that “The overall referral rate for the screening program across the 4 years was 11.1% but referral rates decreased by between 2 and 4% for each year of program existence with a 5% rate in year 4. Only 32% of the rescreens were completed at the hospital, and no data was available for the remaining infants because parents were provided a choice of follow up centers. Referral for a diagnostic assessment after the rescreens at the hospital was predictive of sensorineural hearing loss in one-third of cases and the estimated prevalence was 3 per 1000”. Due to the large loss of subjects to be retested by ABR, this value of prevalence underestimates the true HL incidence value in the Gauteng area.

The third paper by Storbeck and Pittman [25] appeared in the 2008 special issue of the International Journal of Audiology, dedicated to the early detection and hearing intervention (EDHI) programs in Africa. The authors reported data “from a family-centred, home-based intervention program (HI HOPES) over a 12-month period in order to track the effectiveness of the holistic unbiased support to families of infants and toddlers with a hearing-loss”. The SNHL of the children was classified as “moderate loss 2 cases, moderate to severe loss 4 cases, moderate to profound loss 3 cases, severe to profound loss 7 cases and profound loss 15 cases”. For the 32 children who participated in the program, 24 received a digital hearing aid, 3 a cochlear implant; for 1 infant, the family declined amplification, and for another, it was not possible to amplify due to an ear malformation. Obviously, the structure of the project did not permit an estimate of the HL incidence in the assessed areas.

The fourth paper by Bezuidenhoutet al. [22] assessed the challenge of applying a UNHS model in a public context in Johannesburg, South Africa. The project used a double (i.e., test and retest) OAE protocol with TEOAEs or DPOAEs and a clinical ABR for diagnosis. The authors report that “of 2740 neonates born during the study period, 490 (17.9%) were identified for screening, and distortion product otoacoustic emissions screening was conducted on 121 (4.4%). Repeat screening was required in 57 (47.1%) neonates, but only 20 returned for follow-up”. The most important challenges to the feasibility of UNHS implementation were the insufficient number of audiologists available to provide screening, the high rate of false positive test results, and the unacceptably high rates of loss to follow-up. Two modifiable factors, namely the presence of vernix caseosa in the external ear canal and high ambient noise levels, were found to have significantly influenced the screening process.

### 3.9. Uganda

There is no NHS or UNHS in Uganda, and the only information from hearing screening practices comes from two recent papers by Seguya et al. in 2021 [25] and Ndoleriire et al. in 2022 [26]. For both papers, TEOAEs were used for testing and retesting. ABR was used diagnostically in the second phase of the assessment.

In the first paper by Seguya et al., the authors report that “We recruited infants aged 1 day to 3 months and performed a three-staged screening. At stage 1, we used Transient Evoked Oto-acoustic Emissions (TEOAEs), at stage 2 we repeated TEOAEs for infants who failed TEOAEs at stage 1 and at stage 3, we conducted Automated brainstem responses (ABRs) for those who failed stage 2. Infant Hearing Loss (IHL) was present if they failed an ABR at 35 dBHL”. A total of 401 infants were tested, and 299 passed stage 1, while the remaining 102 (25.4%) were referred for stage 2. Of those, 35 (34.3%) returned for stage 2 screening. Five infants failed the second TEOAE assessment in at least one ear. From those, four were identified with a unilateral SNHL, and one with a bilateral SNHL. The estimated HL incidence was 14.9 per 1000 (5/334). Unfortunately, this high number is an underestimate of the actual incidence, since only 34.3% of the referred infants returned for the ABR evaluation.

In the second paper by Ndoleriire et al., the authors reported the efficacy of hearing screening in children between 0 and 59 months. The data reported refer to a sample of 1217 children of various ages, tested in two phases with TEOAEs. From those, 27 failed the TEOAE test unilaterally and 18 bilaterally. Factors affecting the TEOAE failure included rural residence (*p* = 0.027), a low birth weight (*p* = 0.045), relatives having hearing loss (*p* ≤ 0.001), admission to the hospital after birth (*p* = 0.012), and a history of a childhood suppurative otitis media (*p* = 0.015). No other information on these 45 children is available.

**Table 3:** Data related to the various African NHS national activities. The national data are presented in alphabetical order. The gray shaded estimates indicate high prevalence values: BD = bilateral deafness; WB = well babies; NICU = infants from the intensive care unit; NA = data not available; HA = hearing aid; CI = cochlear implant.

Country	Participants (n)	Screening Protocol	Hearing Loss Prevalence	Causes	Intervention Policies
Cameroon	NA	NA	9–36 per 1000	Non-syndromic hearing impairment Waardenburg and Usher syndromes	NA
Côte d’Ivoire	1495	TEOAE, clinical ABR	5.96 per 1000	NA	NA
Egypt	150	TEOAE, clinical ABR	360 × 1000	Consanguinity risk factor, presence of I71N mutations in the CJB2 gene	NA
Ghana	50	DPOAE	NA	NA	NA
Kenya	9963	DPOAE + AABR, ABR	3 per 1000	NA	NA
Nigeria	4718		NA	Probable auditory neuropathy in 6/13 SNHL cases	NA
	1745	TEOAE + AABR	NA	NA	NA
Sudan	1120		10.8 per 1000 NICU 5.2 per 1000 WB	NA	NA
South Africa	510	DPOAE + 1000 Hz tymp, ABR	NA	NA	NA
	6241	TEOAE, ABR	3 per 1000	NA	NA
	32	NA	NA		HA (24), CI (3)
	490	TEOAE/DPOAE, ABR	NA	NA	NA
Uganda	401 1217	TEOAE + ABR TEOAE + ABR	14.9 per 1000 NA	NA NA	NA NA

## 4. Discussion

The first objective of the paper was to provide updates on the African NHS/UNHS practices, from reports found in the literature in the period 2004–2024. The second objective of the paper was to extract information from the identified ongoing UNHS programs, regarding the coverage of the project, the congenital hearing impairment estimates (with emphasis into the bilateral hearing loss), the description of causes leading to hearing loss and the relative intervention strategies, and lastly, the technologies and protocols used. The sections below summarize the reported data in the assessed 15 manuscripts.

### 4.1. The Number of NHS Programs

In the previous review on the European NHS-UNHS programs [5], we reported that it was quite surprising to discover that the European data on NHS are very poor, and very few reports exist to describe the actual NHS programs in Europe. The African NHS data in the literature are even more scarce. Of the nine African states where there are reports about an NHS program, none has a functional or presently active cross-country NHS/UNHS. Adadey et al. [17] mentions the existence of a UNHS program in Ghana (from the 1970s), which is not only not active across the whole country, but the output data from screening (i.e., intervention) are unknown. Data from Olusanya [8] and Engelman [27] suggest that, in most African states, there are sporadic local hospital-based screening programs, using the established two-phase OAE assessment with a clinical ABR for third-stage case referrals.

### 4.2. Reported NHS/UNHS Coverage: Issues of Loss to Follow-Up

There are no data in the literature regarding NHS coverage, in terms of country territories. According to the data in Table 3, most large studies in Kenya [18], Nigeria [20], and South Africa [24] refer to local activities usually spanning a single hospital territory.

An interesting observation of the data in Table 3 is that, in a number of African hearing screening realities [12,17,20,21,25], the percentage level of the loss to follow-up (LTF) infants is quite high (i.e., >15%), especially for subjects who did not pass the first OAE assessment. Generally, the percentage of LTF is an index of the organizing efficiency of the NHS/UNHS program and in European and other Western countries has been initially attributed to loopholes in the NHS phases, i.e., test, retest, etc. [28,29].

More recent studies in the literature have better explored the innerworkings of LTF and have suggested that a number of social and demographic factors have a negative impact on it [30,31]. Factors contributing to a higher LTF include the following: single maternal marital status (the younger the single mothers the higher the LTF), having an insurance policy, the education level of the mother, and the residence of the mother in a rural or in a city location. These factors can be easily identified in any of the African screening realities. In addition, due to lack of extensive parental information [8,22], the social stigma of having hearing loss [32] possibly increases the LTF percentages. The relationship between LTF and social stigma has not been elucidated completely, and additional studies are needed.

### 4.3. Estimates of Bilateral Hearing Loss Prevalence

The fact that there are no country-wide or large UNHS programs implies that the reported hearing loss incidences are not precise. In many cases, where the authors do not present more detailed data, it is assumed that the reported incidences are an average between NICU infants and well babies.

The highest incidence has been reported in Egypt (360 per 1000) in a rather small sample of selected infants. Other high HL incidences have been reported in Uganda (14.9 per 1000), Sudan (10.8 per 1000 in the NICU), and Cameroon (9–36 per 1000). A note for the latter study using the Cameroon data (Tingtang et al. [11]), the HL prevalence data were assembled not from NHS singular databases but from independent studies in Cameroon, affronting various pathologies that impact hearing.

The studies from both Kenya and Soth Africa (3 per 1000) are closer to the reported norms of European [33] and North American [34] studies and the data released by the World Health Association in 2021, with a prevalence of 3.74 per 1000 [35]. The studies from Kenya and South Africa assessed data from relatively large sample sets (9943 and 6241 cases, respectively), and probably, for this reason, the HL prevalence estimates are closer to the international values. The Nigerian screening reports used large datasets, but unfortunately, the corresponding authors did not provide any HL prevalence data.

### 4.4. Causes Leading to Hearing Loss and Requiring Intervention Strategies

Data in this section are very scarce, since the majority of the papers have only reported screening performance metrics, rather than the HL prevalence and EDHI strategies.

In terms of risk factors leading to possible HL, data from Egypt [14,15] suggest consanguinity and various mutations of the CJB2 gene. Consanguinity as a risk factor has been reported in other papers from Nigeria [19], South Africa [23], and the Sub-Saharan states [27] and probably mirrors African social practices.

In terms of intervention policies, the literature is extremely vague, and only the paper by Storbeck and Pittman [9] discussed hearing aids and cochlear implants in a group of South African children, within the context of the HI HOPES project.

Considering the lack of screening programs and the presence of only sporadic hearing intervention strategies, it can be assumed that the identification of a hearing deficit and a possible intervention occurs late, probably at early-school age. There are data on this topic; see, for example, Skarzynski et al. [36] on the Lagos schoolchildren screening results and Jesuyajolu et al.’s [37] review on the Nigerian cochlear implant data, but more detailed information in this direction is outside the scope of this review.

### 4.5. Technological Issues

All the screening papers report data from a two-stage OAE protocol, where the third stage was an AABR or, directly, a diagnostic ABR. Distortion product otoacoustic emissions were used in the papers from Ghana [16], Kenya [18], and South Africa [23]. There is a lack of information on the actual OAE hardware used, but the descriptive information of these projects refers primarily to portable OAE screeners, which probably have the option to carry out an AABR assessment of the tested infant. In this context, no questions were raised regarding the OAE probe calibration issues that we have postulated in previous papers [38,39] and in the European hearing screening data review [5].

### 4.6. Factors Affecting the NHS Practices in Africa

According to Kanji [36], poor-quality child health services for children who are ill was observed in relation to waiting periods, staff skills, triage, and promotional, prevention, and curative care. This was further accompanied by insufficient developmental assessment. Inconsistency was noticed in terms of immunization and rehabilitation.

Ameyaw et al. [16] pointed out that, because of the lack of hearing healthcare specialists, especially pediatric audiologists, newborn hearing screening and other infant hearing services are accessible only at the Korle-Bu Teaching Hospital (KBTH) located in the Greater Accra Region of Ghana.

Furthermore, Jesuyajolu et al. [37] commented that hearing screening and subsequent interventions in Nigeria are impeded by high cost, lack of full rehabilitative facilities and staff, skepticism, and a lack of funding.

These comments from the clinical realities in South Africa, Ghana, and Nigeria are the background realities of all exposed programs in this review. Economic factors, lack of professional figures, and skepticism related to the stigma of hearing loss are the key players in the resistance to neonatal hearing screening.

Of course, in the last decade, the costs of screening equipment (especially the ones based on OAEs) have decreased significantly, so the key element in the problems listed above is to affront the availability of a professional audiologist. This aspect is much more complicated, because it involves not only educational resources (for the scientific formation of audiology technicians and audiologists) but also a state budget.

One way to address this problem is to develop tele-medicine resources. Various papers in the literature have already addressed not only the feasibility of implementing such a structure in an African reality [16,40,41,42] but also the possibility of lowering the reported LTF percentages [43]. While the concept of tele-audiology is quite attractive, it should be considered that most of the tele-audiology data come from the state of South Africa. Taking into consideration the economic dimensions of the necessary infrastructure [41], the available data in the literature do not specify whether the tele-audiology model can be exported to all rural African realities, as a solution to a more globalized NHS.

### 4.7. Limitations of the Study

It might be possible that some publications in local African journals that are not indexed in PubMed or Scopus were not considered in the filtering process of the manuscript evaluation. On the other hand, such manuscripts (with no impact factor) would challenge the quality criteria we have imposed on this scoping review.

### 4.8. Future Directions

More clinical studies are required to identify and assess the cultural attitudes toward hearing loss and how stigma might influence screening acceptance and follow-up. There is also a need for more information regarding the transition from NHS to EDHI.

This last section was requested by one of the reviewers of the manuscript who posed the interesting question of whether an improvement of hearing screening practices in Africa could generally improve the audiological services to those in need. Theoretically, the answer should have been a “yes”. However, the available data from the African screening policies do not support such a relationship for at least two main reasons, as follows: (1) the level of the audiological services depends heavily on the local economics [28,37] and (2) the availability or rather the lack of audiology professionals per territory [8,16]. The data presented also suggest that the social stigma of deafness and the lack of extensive information to the families [40] might worsen the synergy between an NHS program and other audiological intervention services, causing high levels of loss to follow-up (LFU), discussed in Section 4.2.

In summary, the lack of precise hearing screening and follow-up data results in a lack of a workable model exploring the effects of a successful NHS program on the general audiological services. This sort of information needs to be elucidated as soon as possible.

## 5. Conclusions

The data on the African neonatal hearing screening are quite scarce, and no country-wise programs exist at the moment. The various screening realities are implemented within big urban centers, leaving the residents of rural areas unassisted. For the latter, proposals based on tele-medicine protocols have been suggested.

The data on HL prevalence are also incomplete, but the available data refer to rates from 3 to 360 subjects per 1000. These data cannot be taken at face value but within the small-sample size context in which they were acquired.

Regarding the causes of HL, very few data have reported that consanguinity is the most attributed factor, at least in the Sub-Saharan African states.

## Figures and Tables

**Table 1:** Assessed countries for the presence NHS activities. The cells in gray indicate the countries where hearing screening data have been published.

ID	Country	Population	Region	Screening Program
1	Nigeria	232,679,478	Western Africa	Partial
2	Ethiopia	132,059,767	Eastern Africa	Partial
3	Egypt	116,538,258	Northern Africa	Partial
4	DR Congo	109,276,265	Middle Africa	none
5	Tanzania	68,560,157	Eastern Africa	none
6	South Africa	64,007,187	Southern Africa	Partial
7	Kenya	56,432944	Eastern Africa	Partial
8	Sudan	50,448,963	Northern Africa	none
9	Uganda	50,015,092	Eastern Africa	Partial
10	Algeria	46,814,308	Northern Africa	none
11	Morocco	38,081,173	Northern Africa	none
12	Angola	37,885,849	Middle Africa	none
13	Mozambique	34,631,766	Eastern Africa	none
14	Ghana	34,427,414	Western Africa	Partial
15	Madagascar	31,964,956	Eastern Africa	none
16	Côte d’Ivoire	31,934,230	Western Africa	Partial
17	Cameroon	29,123,744	Middle Africa	none
18	Niger	27,032,412	Western Africa	none
19	Mali	24,478,595	Western Africa	none
20	Burkina Faso	23,548,781	Western Africa	none
21	Malawi	21,655,286	Eastern Africa	none
22	Zambia	21,314,956	Eastern Africa	none
23	Chad	20,299,123	Middle Africa	none
24	Somalia	19,009,151	Eastern Africa	none
25	Senegal	18,501,984	Western Arica	none

**Table 2:** The 15 eligible papers after the filtering process. The national data are presented in alphabetical order. NA% implies that the data for the population coverage were not available (as in the case of isolated local studies).

n	Country	Sample Size (n)	Year the Study Was Carried Out, Population % Coverage	Authors	Publication Year
1	Cameroon	NA	NA%	Tingang et al.	2020
2	Cote d’Ivoire	Pilot study 1495	July 2007 to March 2008 NA%	Tanon-Anoh et al.	2009
3	Egypt	150	2009 and March 2010 NA%	Imam et al.	2013
4	Ghana	50	NA, NA%	Ameyaw et al.	2019
5	Kenya	9963	NA, NA%	Ndegwa et al.	2024
6	Nigeria	1745	July 2005 to December 2006, NA%	Olusanya et al.	2009
7		4718	NA, NA%	Olusanya et al.	2010
8	Sudan	1120	Feb 2014 to Feb 2019 NA%	Kardman et al.	2023
9	South Africa	510	5 months, NA%	Swanepoel et al.	2006
10		6241	4 y, NA%	Swanepoel et al.	2007
11		32	NA%	Storbeck and Pittman	2008
12		490	NA, NA%	Bezuidenhout et al.	2018
13	Uganda	401	NA, NA%	Seguya et al.	2021
14		1217	NA, NA%	Ndoleriire et al.	2022
15	Sub-Saharan area	NA	NA, NA%	Engelman	2014

## Data Availability

Data available upon request.

## Supplementary Materials

The following supporting information can be downloaded at: https://www.mdpi.com/article/10.3390/children12020141/s1, Table S1: PRISMA 2020 Checklist [44].

## Abbreviations

AABR	Automated auditory brainstem response
ABR	Auditory brainstem response
BD	Bilateral deafness
EDHI	Early hearing detection and intervention
NICU	Neonatal intensive care unit
OAE	Otoacoustic emissions
UNHS	Universal neonatal hearing screening
